# Metformin Inhibits Advanced Glycation End Products-Induced Inflammatory Response in Murine Macrophages Partly through AMPK Activation and RAGE/NF*κ*B Pathway Suppression

**DOI:** 10.1155/2016/4847812

**Published:** 2016-09-28

**Authors:** Zhong'e Zhou, Yong Tang, Xian Jin, Chengjun Chen, Yi Lu, Liang Liu, Chengxing Shen

**Affiliations:** ^1^Department of Cardiology, Xinhua Hospital, Shanghai Jiao Tong University School of Medicine, 1665 Kongjiang Road, Shanghai 200092, China; ^2^Department of Cardiology, Central Hospital of Minhang District, 170 Xinsong Road, Shanghai 201199, China

## Abstract

Advanced glycation end products (AGEs) are major inflammatory mediators in diabetes, affecting atherosclerosis progression via macrophages. Metformin slows diabetic atherosclerosis progression through mechanisms that remain to be fully elucidated. The present study of murine bone marrow derived macrophages showed that (1) AGEs enhanced proinflammatory cytokines (interleukin-1*β* (IL-1*β*), IL-6, and tumor necrosis factor-*α* (TNF-*α*)) mRNA expression, RAGE expression, and NF*κ*B activation; (2) metformin pretreatment inhibited AGEs effects and AGEs-induced cluster designation 86 (CD86) (M1 marker) expression, while promoting CD206 (M2 marker) surface expression and anti-inflammatory cytokine (IL-10) mRNA expression; and (3) the AMPK inhibitor, Compound C, attenuated metformin effects. In conclusion, metformin inhibits AGEs-induced inflammatory response in murine macrophages partly through AMPK activation and RAGE/NF*κ*B pathway suppression.

## 1. Introduction

Atherosclerosis, one of the main complications of diabetes, constitutes the leading cause of morbidity and mortality in today's world. Macrophages, as major inflammatory contributors, are key modulators of atherosclerotic plaque formation and progression [1]: M1 macrophages, which are related to inflammatory effects, stimulate inflammation and promote plaque progression, while M2 macrophages, which link with anti-inflammatory roles, contribute to inflammation resolution and atheroma regression [2, 3]. Previous studies have demonstrated that excessive inflammatory M1 monocytes/macrophages emerge in peripheral blood of both prediabetic and diabetic patients [4, 5]. Furthermore, our previous study also found that M1 monocytes/macrophages, the inflammatory subset, increased in circulation and atherosclerotic plaque in STZ-induced diabetic mice and were related to enhanced inflammation and accelerated atherosclerosis [6]. Thus, the increased inflammatory macrophages in diabetes contribute to persistent low grade inflammation and accelerated atherosclerosis.

Various factors are responsible for macrophage inflammatory activation in diabetes. Besides hyperglycemia, the advanced glycation end products (AGEs), which are generated irreversibly in high glucose condition, are another group of critical pathogenic factors in diabetes. Previous studies showed AGEs driven inflammatory response in macrophages [7] and promoted atherosclerosis [8]. Because inflammatory macrophages dominate the progression of atherosclerosis, blockage or attenuation of AGEs-induced macrophage inflammatory response might alleviate diabetic atherosclerosis.

In addition to its antihyperglycemic effects, metformin, with over half-decade use as first-line therapy for type 2 diabetes [9], slows down diabetic atherosclerosis development [10] through mechanisms that are still not fully understood. Recently, metformin's anti-inflammatory properties were demonstrated in lipopolysaccharide induced macrophages [11, 12], and previous studies had shown that metformin could downregulate RAGE expression [13] and suppress NF*κ*B signaling in various cells [14, 15]. As our previous work proved that RAGE/NF*κ*B signaling was involved in AGEs-induced macrophage inflammatory activation [7], it is reasonable to hypothesize that metformin might inhibit AGEs-induced inflammatory response in macrophages. Therefore, the present study tested the hypothesis and could provide novel elucidation for the antiatherosclerotic effect of metformin.

## 2. Materials and Methods

### 2.1. Mice

Male C57BL/6 mice (8 weeks old) were purchased from Slac Laboratory Animal Co., Ltd. (Shanghai, China). The mice were housed under specific pathogen-free conditions with controlled temperature (22–25°C) and 12 h light/dark cycles; they were given standard chow and water ad libitum. All animal experiments were approved by the Ethics Committee of Xinhua Hospital Affiliated to Shanghai Jiao Tong University School of Medicine (approval number XHEC-F-2016-012).

### 2.2. Preparation and Culture of Bone Marrow Derived Macrophages (BMDMs)

Mice were anesthetized with pentobarbital (50 mg/kg, i.p.) to minimize suffering and killed by cervical dislocation without recovery from anesthesia. Tibias and femurs were removed and bone ends were cut off, and bone marrow was flushed with PBS supplemented with 1% fetal bovine serum (FBS, Gibco, Australia, Cat# 10099-141). A single cell suspension was prepared by filtering the cells through a 40 *μ*m strainer (BD Falcon, Cat# 352340). The cell suspension was centrifuged at 1500 rpm for 5 min, and the cell pellet was resuspended in culture medium. Bone marrow cells were cultured for 7 days at 37°C with 5% CO_2_ in Dulbecco's modified Eagle's medium (DMEM), high glucose (HyClone, Beijing, China, Cat# SH30022.01B) supplemented with 10% FBS, 2 mM L-glutamine (Sigma-Aldrich, Missouri, USA, Cat# 59202C), 100 U/mL penicillin and 100 *μ*g/mL streptomycin (Beyotime, Jiangsu, China, Cat# C0222), and 20 ng/mL GM-CSF (PeproTech, New Jersey, USA, Cat# 315-03). Cells were harvested on day 7 for further experiments. Macrophages (>95%) were identified by flow cytometry with anti-CD11b APC and anti-F4/80 FITC (eBioscience, California, USA, Cat# 17-0112 and Cat# 11-4801) staining.

BMDMs were seeded at a density of 0.5 × 10^6^/mL and cultured overnight before stimulation. Based on experimental protocol, the BMDMs were treated with different concentrations of metformin (0.25, 1.0, and 2.0 *μ*M) or AGEs (200 mg/L) for different time periods. For pathway studies, the BMDMs were pretreated with anti-RAGE antibody or ammonium pyrrolidinedithiocarbamate (PDTC) or Compound C for 60 min. The anti-RAGE neutralizing antibody (R&D Systems, Minnesota, USA, Cat# AF1179) was reconstituted at 0.2 mg/mL in sterile PBS and further diluted with culture medium to the final concentration of 20 *μ*g/mL. Metformin hydrochloride (Abcam, Cambridge, UK, Cat# ab120847) was dissolved in water at a concentration of 50 mM and further diluted with culture medium to the final concentrations (0.25, 1.0, and 2.0 *μ*M). The NF*κ*B inhibitor PDTC (Abcam, Cambridge, UK, Cat# ab141406) was dissolved in DMSO at a concentration of 100 mM and further diluted with culture medium to the final concentration of 50 *μ*M (contains 0.5% DMSO, v/v). The AMPK inhibitor Compound C (Abcam, Cambridge, UK, Cat# ab120843) was firstly dissolved in DMSO at a concentration of 10 mM and further diluted with culture medium to the final concentration of 5 *μ*M (contains 0.5% DMSO, v/v). To rule out the influence of DMSO, proper dosages of DMSO were added to culture medium to meet the groups with the highest concentration of DMSO. AGE-BSA was purchased from Anyan-bio Technology (Shanghai, China, Cat# AY-4710P), and the corresponding amount of BSA was used as control.

### 2.3. Real-Time PCR Analysis

Total RNA was extracted from BMDMs using TRIzol reagent (Takara, Liaoning, China, Cat# 9109), and 1 *μ*g of total RNA was reverse transcribed to cDNA using the PrimeScript RT Master Mix kit (Takara, Liaoning, China, Cat# RR036A). Real-time PCR array analysis was performed by using the SYBR Premix Ex Taq*™* kit (Takara, Liaoning, China, Cat# RR420A) in a total volume of 20 *μ*L, with 2 *μ*L of cDNA primers (0.2 mM each), 10 *μ*L of SYBR Green, and 0.4 *μ*L of Rox Dye II. The standard PCR conditions consisted of 95°C for 30 sec, followed by 40 cycles of 95°C for 5 sec and 60°C for 34 sec, with a final dissociation stage; the samples were run on an ABI 7500 detector (Applied Biosystems, California, USA). The amounts of target genes were determined and normalized to the amount of GAPDH cDNA (Sangon Biotech, Shanghai, China, Cat# B661304). The sequences of the primers (synthesized by Sangon Biotech, Shanghai, China) for the target genes were as follows: Fwd 5′-CTCACAAGCAGAGCACAAGC-3′ and Rev 5′-TCCAGCCCATACTTTAGGAAGA-3′ for IL-1*β*; Fwd 5′-TCTGCAAGAGACTTCCATCCA-3′ and Rev 5′-AGTCTCCTCTCCGGACTTGT-3′ for IL-6; Fwd 5′-GGTGCCTATGTCTCAGCCTC-3′ and Rev 5′-CCACTTGGTGGTTTGTGAGTG-3′ for TNF-*α*; Fwd 5′-TGCACTACCAAAGCCACAAG-3′ and Rev 5′-TGATCCTCATGCCAGTCAGT-3′ for IL-10.

### 2.4. Western Blot Analysis

After different stimulations, the BMDMs were collected and cellular lysates were prepared. Equal amounts of protein (40–60 *μ*g) were resolved on SDS-PAGE and transferred to polyvinyl difluoride (PVDF) membranes. The blots were blocked with 5% milk in TBST for 1 h at room temperature and then incubated with primary rabbit anti-mouse antibodies overnight at 4°C. The antibodies against p-AMPK (1 : 1000, Cat# ab133448) and RAGE (1 : 1000, Cat# ab3611) were purchased from Abcam (Cambridge, UK), and the antibodies against NF*κ*B-65 (1 : 1000, Cat# 8242S) and p-NF*κ*B-65 (1 : 1000, Cat# 3033S) were purchased from Cell Signaling Technology (Massachusetts, USA). The blots were subsequently incubated with the secondary goat anti-rabbit antibodies conjugated with horseradish peroxidase (1 : 1000) for one hour and enhanced using a chemiluminescence system (ChemiDoc XRS+, Bio-Rad Laboratories, USA). The intensity of each band was normalized to the loading control tubulin (Beyotime, Jiangsu, China, Cat# AT819).

### 2.5. Flow Cytometry Analysis

After different stimulations, single cell suspensions of BMDMs were prepared in flow buffer and incubated with antibodies. Anti-mouse CD86 PE (eBioscience, California, USA, Cat# 12-0862) was used to identify M1 macrophages while anti-mouse CD206 FITC (BioLegend, California, USA, Cat# 141704) was used to identify M2 macrophages. Results were acquired with a BD Canto II flow cytometer (BD Biosciences, USA) and analyzed by the FlowJo software (Tree Star, USA).

### 2.6. Immunofluorescent Staining

BMDMs were plated in 24-well plates. After different stimulations, cells were washed thrice with cold PBS and fixed in 4% (w/v) paraformaldehyde for 20 min and then washed with PBS again. Next, cells were incubated in buffered normal goat serum to prevent nonspecific binding of antibodies for 1 h at room temperature. They then were incubated overnight with antibody against NF*κ*B-65 (1 : 200, Cat# 8242S) purchased from Cell Signaling Technology (Massachusetts, USA), followed by incubation with Cy3 goat anti-rabbit IgG (1 : 500; Beyotime, Cat# A0516) for 1 h at 37°C. Thereafter, cells were washed in PBS. DAPI was used to stain the cell nuclei (blue) at a concentration of 1.43 *μ*M (Sigma, St. Louis, USA, Cat# D8417). Photomicrographs were taken with a Leica DMI3000B camera (Leica, Germany).

### 2.7. Statistical Analysis

All results are expressed as mean ± SD. One-way analysis of variance (ANOVA) was used to assess the effects of one factor among multiple groups, and post hoc testing was done by Tukey test. Two-way ANOVA was used to assess the effects of two factors among multiple groups, and post hoc testing was done by Bonferroni test. Analysis was performed using SPSS software 19.0 (SPSS Inc., Chicago, USA) for Windows. A two-tailed value of *p* < 0.05 was considered statistically significant.

## 3. Results

### 3.1. AGEs-Induced Inflammatory Response in BMDMs through RAGE/NF*κ*B Signaling

**Figure 1:**
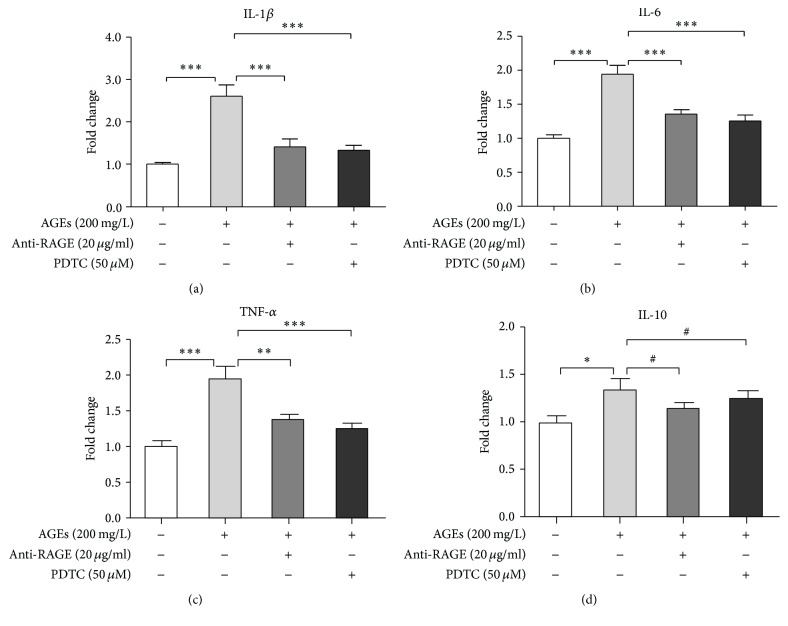
AGEs-induced inflammatory response in BMDMs through RAGE/NF*κ*B signaling. BMDMs were divided into 4 groups: control, AGEs, AGEs + anti-RAGE, and AGEs + PDTC group. Cells in the last two groups were pretreated with anti-RAGE antibody (20 *μ*g/mL) or PDTC (50 *μ*M) for 60 min, respectively, and then, together with AGEs group, the three groups were cultured with AGEs (200 mg/L) for 24 h. The control group was treated with BSA (200 mg/L) for the same amount of time. RNA then was extracted, and mRNA levels of IL-1*β* (a), IL-6 (b), TNF-*α* (c), and IL-10 (d) were measured by real-time PCR. Bar graphs represent the results (mean ± SD) of three independent experiments. One-way ANOVA was applied and all the overall ANOVA was significant. ^#^ *p* > 0.05; ^*∗*^ *p* < 0.05; ^*∗∗*^ *p* < 0.01; and ^*∗∗∗*^ *p* < 0.001 when compared between selected groups.

Our previous study elucidated that AGEs promoted BMDMs to express proinflammatory cytokines through RAGE/NF*κ*B signaling [7]. To confirm this finding, here we tested cytokine expression profile of BMDMs after AGEs stimulation with or without anti-RAGE neutralizing antibody or PDTC pretreatment. BMDMs were divided into 4 groups: control, AGEs, AGEs + anti-RAGE, and AGEs + PDTC group. Cells in the last two groups were pretreated with anti-RAGE antibody (20 *μ*g/mL) or PDTC (50 *μ*M) for 60 min, respectively, and then, together with AGEs group, the three groups were cultured with AGEs (200 mg/L) for 24 h. The control group was treated with BSA (200 mg/L) for the same amount of time. mRNA levels of proinflammatory cytokines (IL-1*β*, IL-6, and TNF-*α*) and anti-inflammatory cytokine (IL-10) were measured by real-time PCR.  Figure 1 shows that AGEs markedly increased mRNA expression of proinflammatory cytokines (IL-1*β*, IL-6, and TNF-*α*) (Figures 1(a), 1(b), and 1(c)), while only slightly upregulating that of IL-10 (Figure 1(d)), indicating that AGEs predominantly induced inflammatory response in murine macrophages. In addition, pretreatment with anti-RAGE antibody or PDTC ameliorated the proinflammatory effects of AGEs (Figures 1(a), 1(b), and 1(c)). Therefore, RAGE/NF*κ*B signaling is involved in AGEs-induced inflammatory response in macrophages.

### 3.2. Metformin Inhibited AGEs-Induced Inflammatory Response in BMDMs

**Figure 2:**
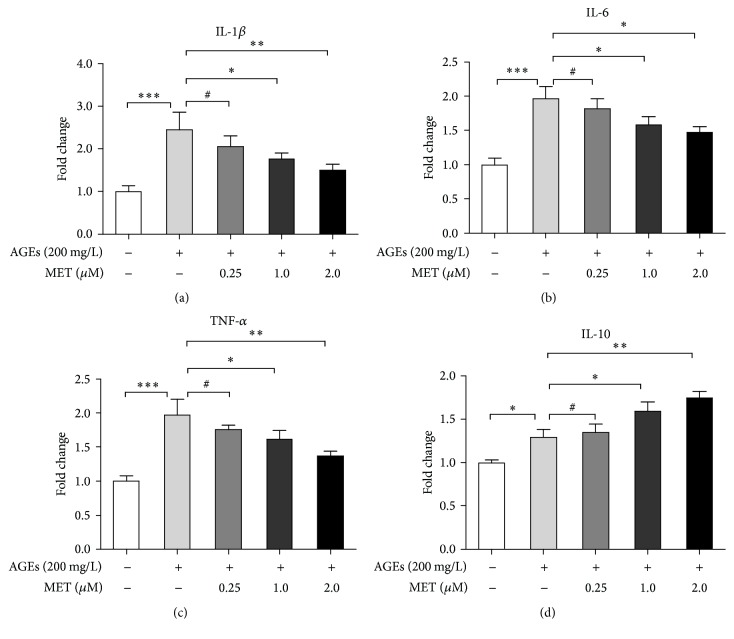
Metformin inhibited AGEs-induced inflammatory response in BMDMs. BMDMs were divided into 5 groups: control, AGEs, AGEs + MET 0.25 (metformin 0.25 *μ*M), AGEs + MET 1.0 (metformin 1.0 *μ*M), and AGEs + MET 2.0 (metformin 2.0 *μ*M). Cells in the last 3 groups were pretreated with different concentrations of metformin (0.25, 1.0, and 2.0 *μ*M) for 60 min, respectively, and then, together with AGEs group, the 4 groups were stimulated with AGEs (200 mg/L) for 24 h. The control group was treated with BSA (200 mg/L) for the same amount of time. RNA then was extracted, and mRNA levels of IL-1*β* (a), IL-6 (b), TNF-*α* (c), and IL-10 (d) were measured by real-time PCR. Bar graphs represent the results (mean ± SD) of three independent experiments. One-way ANOVA was applied and all the overall ANOVA was significant. ^#^ *p* > 0.05; ^*∗*^ *p* < 0.05; ^*∗∗*^ *p* < 0.01; and ^*∗∗∗*^ *p* < 0.001 when compared between selected groups.

Because metformin has anti-inflammatory potential [11, 12], next we tested whether metformin inhibited AGEs-induced inflammatory response in macrophages. BMDMs were divided into 5 groups: control, AGEs, AGEs + MET 0.25 (metformin 0.25 *μ*M), AGEs + MET 1.0 (metformin 1.0 *μ*M), and AGEs + MET 2.0 (metformin 2.0 *μ*M). Cells in the last 3 groups were pretreated with different concentrations of metformin (0.25, 1.0, and 2.0 *μ*M) for 60 min, respectively, and then, together with AGEs group, the 4 groups were stimulated with AGEs (200 mg/L) for 24 h. The control group was treated with BSA (200 mg/L) for the same amount of time. mRNA levels of proinflammatory cytokines (IL-1*β*, IL-6, and TNF-*α*) and anti-inflammatory cytokine (IL-10) were measured again by real-time PCR.  Figure 2 shows that metformin dose dependently inhibited AGEs' enhancement on mRNA expression of proinflammatory cytokines (Figures 2(a), 2(b), and 2(c)), while promoting that of IL-10 (Figure 2(d)), indicating that metformin inhibited AGEs-induced inflammatory response in BMDMs. As the results showed that 2.0 *μ*M metformin had the strongest effects on downregulating genes expressions of proinflammatory cytokines but upregulating mRNA expression of anti-inflammatory cytokine, we administrated 2.0 *μ*M metformin for the following experiments.

### 3.3. Suppression of RAGE/NF*κ*B Signaling Responsible for Metformin's Inhibition on Inflammatory Response in Macrophages

**Figure 3:**
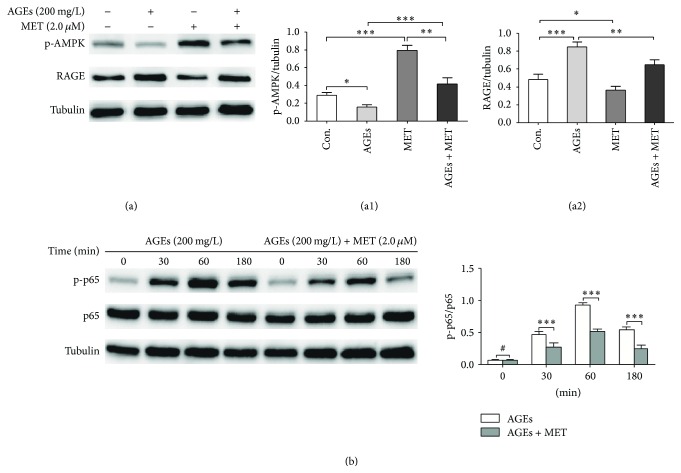
Metformin activates AMPK and inhibits AGEs-induced RAGE expression and NF*κ*B activation. (a) BMDMs were divided into 4 groups: control, AGEs, MET, and AGEs + MET group. In AGEs group, cells were cultured with AGEs at 200 mg/L for 24 h; in MET group, cells were cultured with metformin at 2.0 *μ*M for 24 h; in AGEs + MET group, cells were pretreated with metformin for 60 min and then cultured with AGEs at 200 mg/L for 24 h; in control group, cells were cultured with BSA at 200 mg/L for 24 h. Western blot analysis was performed to measure protein levels of RAGE and phosphorylated AMPK (p-AMPK). Tubulin was used as internal control. (b) BMDMs were pretreated with or without metformin (2.0 *μ*M) for 60 min before AGEs (200 mg/L) stimulation for different time intervals (0, 30, 60, and 180 min). Protein levels of NF*κ*B-p65 (p65) and phosphorylated NF*κ*B-p65 (p-p65) were measured by western blot. Tubulin was used as internal control. Bar graphs represent the results (mean ± SD) of three independent experiments. One-way ANOVA was applied and all the overall ANOVA was significant. ^#^ *p* > 0.05; ^*∗*^ *p* < 0.05; ^*∗∗*^ *p* < 0.01; and ^*∗∗∗*^ *p* < 0.001 when compared between selected groups.

Next, we tested whether inhibition of RAGE/NF*κ*B pathway was responsible for metformin's suppressive effects on AGEs-induced inflammation. Because metformin is an agonist of AMPK and activation of AMPK can abate inflammation in various cells [14, 16, 17], we also evaluated activity of AMPK. Firstly, BMDMs were divided into 4 groups: control, AGEs, MET, and AGEs + MET group. In AGEs group, cells were cultured with AGEs at 200 mg/L for 24 h; in MET group, cells were cultured with metformin at 2.0 *μ*M for 24 h; in AGEs + MET group, cells were pretreated with metformin at 2.0 *μ*M for 60 min and then cultured with AGEs at 200 mg/L for 24 h; in control group, cells were cultured with BSA at 200 mg/L for 24 h. Western blot analysis was performed to measure protein levels of RAGE and phosphorylated AMPK (p-AMPK).  Figure 3(a) shows that AGEs significantly upregulated expression of RAGE and reduced levels of p-AMPK, while metformin had the opposite effect. Moreover, pretreatment with metformin significantly attenuated effects of AGEs on RAGE upregulation and AMPK inactivation. Then, we evaluated the effect of AGEs on activation of NF*κ*B pathway in macrophages with or without metformin (2.0 *μ*M) pretreatment for 60 min before AGEs (200 mg/L) stimulation for different time intervals (0, 30, 60, and 180 min). In western blot analysis (Figure 3(b)), the p-p65/p65 ratio in the AGEs group markedly increased after AGEs stimulation, peaking at 60 min and decreasing thereafter, indicating that NF*κ*B was activated after AGEs stimulation. Furthermore, the p-p65/p65 ratio in the AGEs + MET group was much lower at each time point than in the AGEs group, suggesting that AGEs-induced NF*κ*B activation was partly inhibited by metformin pretreatment. Taken together, these findings suggested that metformin inhibited RAGE/NF*κ*B signaling.

**Figure 4:**
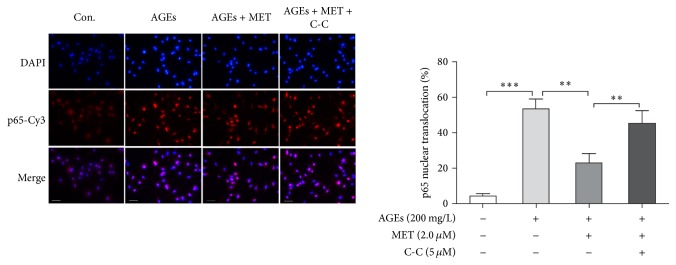
Metformin's inhibition on AGEs-induced NF*κ*B signaling is AMPK dependent. BMDMs were divided into 4 groups: control, AGEs, AGEs + MET, and AGEs + MET + CC group. In AGEs group, cells were cultured with AGEs at 200 mg/L for 60 min; in AGEs + MET group, cells were pretreated with metformin for 60 min and then cultured with AGEs at 200 mg/L for 60 min; in AGEs + MET + C-C group, cells were pretreated with Compound C, an AMPK inhibitor, at 5 *μ*M for 60 min, and then they were treated with metformin at 2.0 *μ*M for 60 min followed by AGEs at 200 mg/L for 60 min; in control group, cells were cultured with BSA at 200 mg/L for 60 min. p65 nuclear translocation of each group was evaluated by immunofluorescent staining. Primary antibodies against p65 and Cy3 (red) labeled secondary antibodies were used to detect p65; DAPI (blue) was used to stain the nucleus. Bar graphs represent the results (mean ± SD) of three independent experiments. Bar = 50 *μ*m. One-way ANOVA was applied and the overall ANOVA was significant. ^*∗∗*^ *p* < 0.01 and ^*∗∗∗*^ *p* < 0.001 when compared between selected groups.

Because metformin is an AMPK activator, we next performed experiments to confirm whether metformin's suppression of NF*κ*B signaling is AMPK dependent. BMDMs were divided into 4 groups: control, AGEs, AGEs + MET, and AGEs + MET + CC group. Because AGEs-induced NF*κ*B activation peaked at 60 min after AGEs stimulation, the duration of AGEs stimulation was set to 60 minutes. In AGEs group, cells were cultured with AGEs at 200 mg/L for 60 min; in AGEs + MET group, cells were pretreated with metformin for 60 min and then cultured with AGEs at 200 mg/L for 60 min; in AGEs + MET + C-C group, cells were pretreated with Compound C, an AMPK inhibitor, at 5 *μ*M for 60 min, and then they were treated with metformin at 2.0 *μ*M for 60 min followed by AGEs at 200 mg/L for 60 min; in control group, cells were cultured with BSA at 200 mg/L for 60 min. p65 nuclear translocation was analyzed by immunofluorescent staining.  Figure 4 shows that p65 nuclear translocation in AGEs group was significantly higher relative to control group indicating NF*κ*B activation; metformin pretreatment significantly inhibited p65 nuclear translocation; and Compound C pretreatment abolished metformin's effects. Therefore, the results evidenced that metformin's inhibition on NF*κ*B was AMPK dependent.

**Figure 5:**
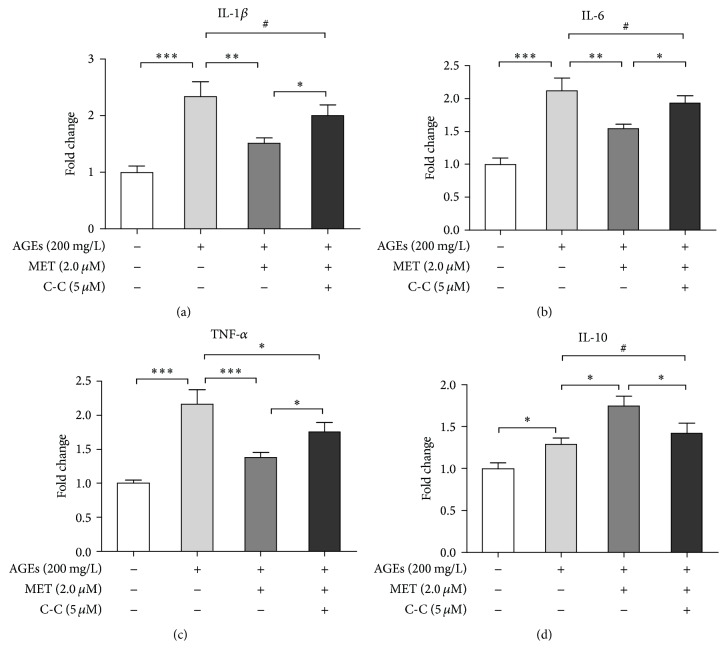
Metformin's inhibition on AGEs-induced inflammatory response is AMPK dependent. BMDMs were divided into 4 groups: control, AGEs, AGEs + MET, and AGEs + MET + CC group. In AGEs group, cells were cultured with AGEs at 200 mg/L for 60 min; in AGEs + MET group, cells were pretreated with metformin for 60 min and then cultured with AGEs at 200 mg/L for 60 min; in AGEs + MET + C-C group, cells were pretreated with Compound C, an AMPK inhibitor, at 5 *μ*M for 60 min, and then they were treated with metformin at 2.0 *μ*M for 60 min followed by AGEs at 200 mg/L for 60 min; in control group, cells were cultured with BSA at 200 mg/L for 60 min. RNA then was extracted, and mRNA levels of IL-1*β* (a), IL-6 (b), TNF-*α* (c), and IL-10 (d) were measured by real-time PCR. Bar graphs represent the results (mean ± SD) of three independent experiments. One-way ANOVA was applied and all the overall ANOVA was significant. ^#^ *p* > 0.05; ^*∗*^ *p* < 0.05; ^*∗∗*^ *p* < 0.01; and ^*∗∗∗*^ *p* < 0.001 when compared between selected groups.

Subsequently, we tested whether metformin's anti-inflammatory effect on AGEs-induced macrophages was dependent upon AMPK activation. BMDMs were divided into 4 groups: control, AGEs, AGEs + MET, and AGEs + MET + C-C group. In AGEs group, cells were cultured with AGEs at 200 mg/L for 24 h; in AGEs+MET group, cells were pretreated with metformin for 60 min and then cultured with AGEs at 200 mg/L for 24; in AGEs + MET + C-C group, cells were pretreated with Compound C at 5 *μ*M for 60 min, and then they were treated with metformin at 2.0 *μ*M for 60 min followed by AGEs at 200 mg/L for 24 h; in control group, cells were cultured with BSA at 200 mg/L for the same amount of time. mRNA levels of proinflammatory cytokines (IL-1*β*, IL-6, and TNF-*α*) and anti-inflammatory cytokine (IL-10) were tested again by real-time PCR.  Figure 5 demonstrated that pretreatment with Compound C attenuated metformin's inhibition on proinflammatory cytokines mRNA expression and promotion of IL-10 mRNA expression in macrophages. Thus, these data indicated that the anti-inflammatory function of metformin was at least partly dependent on AMPK activation.

### 3.4. Metformin Changes AGEs-Induced Surface Markers Expression on Macrophages

**Figure 6:**
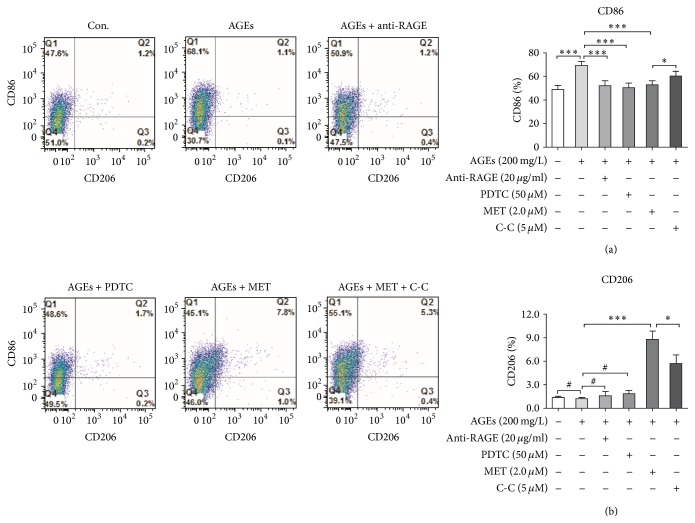
Metformin changes AGEs-induced surface markers expression on macrophages. BMDMs were divided into 6 groups: control, AGEs, AGEs + anti-RAGE, AGEs + PDTC, AGEs + MET, and AGEs + MET + C-C. In AGEs group, cells were cultured with AGEs at 200 mg/L for 24 h; in AGEs + anti-RAGE group, cells were pretreated with anti-RAGE neutralizing antibodies for 60 min followed by AGEs at 200 mg/L for 24 h; in AGEs + PDTC group, cells were pretreated with PDTC for 60 min followed by AGEs at 200 mg/L for 24 h; in AGEs + MET group, cells were pretreated with metformin at 2.0 *μ*M for 60 min and then cultured with AGEs at 200 mg/L for 24 h; in AGEs + MET + C-C group, cells were pretreated with Compound C at 5 *μ*M for 60 min, and then they were treated with metformin at 2.0 *μ*M for 60 min followed by AGEs at 200 mg/L for 24 h; in control group, cells were cultured with BSA at 200 mg/L for the same amount of time. Single cell suspensions then were prepared. M1 surface marker CD86 and M2 surface marker CD206 were detected by flow cytometry analysis. Bar graphs represent the results (mean ± SD) of five independent experiments. One-way ANOVA was applied and all the overall ANOVA was significant. ^#^ *p* > 0.05; ^*∗*^ *p* < 0.05; and ^*∗∗∗*^ *p* < 0.001 when compared between selected groups.

M1 or M2 macrophages are, respectively, considered as pro- and anti-inflammatory macrophages [18]. Because CD86 is one of the important surface markers for M1 (proinflammatory) macrophages and CD206 for M2 (anti-inflammatory) macrophages [19], we next studied metformin's impact on AGEs-induced surface markers (CD86 and CD206) expression on macrophages by flow cytometry analysis. BMDMs were divided into 6 groups: control, AGEs, AGEs + anti-RAGE, AGEs + PDTC, AGEs + MET, and AGEs + MET + C-C. In AGEs group, cells were cultured with AGEs at 200 mg/L for 24 h; in AGEs + anti-RAGE group, cells were pretreated with anti-RAGE neutralizing antibodies for 60 min followed by AGEs at 200 mg/L for 24 h; in AGEs + PDTC group, cells were pretreated with PDTC for 60 min, followed by AGEs at 200 mg/L for 24 h; in AGEs + MET group, cells were pretreated with metformin at 2.0 *μ*M for 60 min and then cultured with AGEs at 200 mg/L for 24 h; in AGEs + MET + C-C group, cells were pretreated with Compound C at 5 *μ*M for 60 min, and then they were treated with metformin at 2.0 *μ*M for 60 min followed by AGEs at 200 mg/L for 24 h; in control group, cells were cultured with BSA at 200 mg/L for the same amount of time. Single cell suspensions then were prepared. M1 surface marker CD86 and M2 surface marker CD206 were detected by flow cytometry analysis. As expected, AGEs significantly increased CD86 but did not affect CD206 expression; and pretreatment with anti-RAGE antibody or PDTC significantly abated AGEs' effect. Pretreatment with metformin partly reversed AGEs' effects on CD86 expression and markedly increased CD206 expression; however, Compound C abated metformin's potency (Figure 6).

## 4. Discussion

The present study provides novel insight into a hypoglycemic agent: metformin inhibits the inflammatory response induced by advanced glycation end products in murine macrophages partly through AMPK activation and suppression of RAGE/NFkappaB signaling.

Macrophage is a vital player in atherosclerosis, with its inflammatory response determining progression of atherosclerotic lesions. AGEs are recognized as strong inflammatory mediators in the diabetic microenvironment, inducing an inflammatory response in macrophages [20, 21]. Therefore, AGEs-induced macrophage activation and inflammation augmentation are critical mechanisms contributing to diabetic accelerated atherosclerosis, rendering it important to inhibit inflammatory response in macrophages in order to slow down or even block atherosclerotic progression. Recently, metformin was shown to have anti-inflammatory effects on lipopolysaccharide (LPS) or PMA induced macrophages [11, 22, 23]. However, it is still unknown whether metformin was able to suppress AGEs-induced inflammatory response in macrophages. The present study showed that pretreatment with metformin not only reduced mRNA expression of proinflammatory cytokines (IL-1*β*, IL-6, and TNF-*α*) but also upregulated mRNA expression of anti-inflammatory cytokine (IL-10) in macrophages. These data evidenced that metformin inhibits AGEs-induced proinflammatory effects in macrophages. Previous study has demonstrated that RAGE/NF*κ*B axis is important in driving inflammation in AGEs-induced macrophages [7]. In the present study, we found that blockade of RAGE or NF*κ*B signaling could significantly attenuate AGEs-induced genes expression of inflammatory cytokines, which confirmed the critical role of RAGE/NF*κ*B signaling in AGEs-induced inflammatory response. In addition, although evidence is mounting that metformin downregulates RAGE expression or inhibits NF*κ*B activity in several cell types [13–15], until the present study, there had been no reports on metformin's inhibition on AGEs-induced RAGE/NF*κ*B signaling in macrophages. Therefore, for the first time, we demonstrated that metformin inhibits AGEs-induced inflammatory response in macrophages via suppressing RAGE/NF*κ*B activation.

Of note, in the present study, we found blockade of RAGE or NF*κ*B signaling or administration of different concentrations of metformin could not completely abolish the proinflammatory effects of AGEs. Such findings hint that pathways other than RAGE/NF*κ*B might also be responsible for AGEs-induced inflammatory response in macrophages. As is well known, in addition to RAGE, AGEs can bind with several other receptors such as AGE-receptor complex, scavenger receptor, and Toll-like receptor 4 (TLR-4) to transmit messages [24, 25]. Furthermore, besides NF*κ*B pathway, MAPK and STAT signaling have been proven to be implicated in driving inflammatory response in macrophages [26, 27]. Therefore, RAGE/NF*κ*B signaling is just one of the many pathways mediating the inflammatory response induced by AGEs; other potential pathways remain to be studied.

Metformin is an AMPK activator, and AMPK signaling suppresses NF*κ*B activation thereby attenuating inflammatory responses [28, 29]. In the present study, pretreatment with Compound C, an AMPK inhibitor, abated metformin's suppression on NF*κ*B as well as proinflammatory cytokines expression, indicating that metformin's inhibition of AGEs-induced inflammatory response in macrophages was AMPK dependent. Of note, in the present study, AGEs slightly increased mRNA expression of IL-10, while pretreatment with metformin significantly strengthened AGEs' induction of IL-10 mRNA expression in a dose dependent manner. In light of the powerful anti-inflammatory function of IL-10, the promotion effect of metformin upon IL-10 expression would further enhance its anti-inflammatory properties. Furthermore, blockade of NF*κ*B signaling did not change IL-10 expression while AMPK blocking treatment significantly ameliorated metformin's promotion on IL-10 expression, indicating that AGEs-induced IL-10 expression was NF*κ*B independent while metformin's enhancement on expression of IL-10 was at least partly AMPK dependent. Therefore, metformin not only suppressed expression of proinflammatory cytokines through AMPK signaling induced NF*κ*B inhibition but also enhanced expression of anti-inflammatory cytokine (IL-10) also via AMPK activation.

A previous study found that metformin primed macrophages into different phenotypes based on the microenvironment [30]. In the present study, metformin inhibited AGEs-induced M1 surface marker CD86 expression while increasing M2 surface marker CD206 expression. Based on the metformin's effects on cytokines expression, results of the present study suggest that metformin probably inhibits AGEs-induced macrophage M1 polarization and might enhance macrophage M2 polarization. Because macrophage polarization determines function, that is, pro- (M1) or anti-inflammatory (M2) [18, 19], the results further support metformin's inhibition of AGEs-induced inflammatory response in macrophages.

## 5. Conclusion

Various metabolic disorders underlie the pathogenesis of diabetic vascular complications. AGEs-induced proinflammatory status of macrophages might be a critical mechanism responsible for diabetic accelerated atherosclerosis. Therefore, attenuating AGEs' proinflammatory effects on macrophages might alleviate atherosclerosis in diabetes. The present study demonstrates that metformin inhibits AGEs-induced inflammatory response in murine macrophages through AMPK activation and suppression of RAGE/NF*κ*B signaling, thereby providing a novel molecular mechanism responsible for metformin's benefits on diabetic atherosclerosis.

## References

[1] Zeller I., Srivastava S. (2014). Macrophage functions in atherosclerosis. *Circulation Research*.

[2] Ley K., Miller Y. I., Hedrick C. C. (2011). Monocyte and macrophage dynamics during atherogenesis. *Arteriosclerosis, Thrombosis, and Vascular Biology*.

[3] Peled M., Fisher E. A. (2014). Dynamic aspects of macrophage polarization during atherosclerosis progression and regression. *Frontiers in Immunology*.

[4] Fadini G. P., Cappellari R., Mazzucato M., Agostini C., Vigili De Kreutzenberg S., Avogaro A. (2013). Monocyte-macrophage polarization balance in pre-diabetic individuals. *Acta Diabetologica*.

[5] Fadini G. P., De Kreutzenberg S. V., Boscaro E. (2013). An unbalanced monocyte polarisation in peripheral blood and bone marrow of patients with type 2 diabetes has an impact on microangiopathy. *Diabetologia*.

[6] Jin X., Liu L., Zhou Z., Ge J., Yao T., Shen C. (2016). Pioglitazone alleviates inflammation in diabetic mice fed a high-fat diet via inhibiting advanced glycation end-product-induced classical macrophage activation. *The FEBS Journal*.

[7] Jin X., Yao T., Zhou Z. (2015). Advanced glycation end products enhance macrophages polarization into M1 phenotype through activating RAGE/NF-B Pathway. *BioMed Research International*.

[8] Prasad A., Bekker P., Tsimikas S. (2012). Advanced glycation end products and diabetic cardiovascular disease. *Cardiology in Review*.

[9] Viollet B., Guigas B., Sanz Garcia N., Leclerc J., Foretz M., Andreelli F. (2012). Cellular and molecular mechanisms of metformin: an overview. *Clinical Science*.

[10] Mamputu J. C., Wiernsperger N. F., Renier G. (2003). Antiatherogenic properties of metformin: the experimental evidence. *Diabetes and Metabolism*.

[11] Kim J., Kwak H. J., Cha J.-Y. (2014). Metformin suppresses lipopolysaccharide (LPS)-induced inflammatory response in murine macrophages via Activating Transcription Factor-3 (ATF-3) induction. *The Journal of Biological Chemistry*.

[12] Bułdak Ł., Łabuzek K., Bułdak R. J. (2014). Metformin affects macrophages' phenotype and improves the activity of glutathione peroxidase, superoxide dismutase, catalase and decreases malondialdehyde concentration in a partially AMPK-independent manner in LPS-stimulated human monocytes/macrophages. *Pharmacological Reports*.

[13] Ishibashi Y., Matsui T., Takeuchi M., Yamagishi S. (2012). Metformin inhibits advanced glycation end products (AGEs)-induced renal tubular cell injury by suppressing reactive oxygen species generation via reducing receptor for AGEs (RAGE) expression. *Hormone and Metabolic Research*.

[14] Gu J., Ye S., Wang S., Sun W., Hu Y. (2014). Metformin inhibits nuclear factor-*κ*B activation and inflammatory cytokines expression induced by high glucose via adenosine monophosphate-activated protein kinase activation in rat glomerular mesangial cells in vitro. *Chinese Medical Journal*.

[15] Hattori Y., Suzuki K., Hattori S., Kasai K. (2006). Metformin inhibits cytokine-induced nuclear factor *κ*B activation via AMP-activated protein kinase activation in vascular endothelial cells. *Hypertension*.

[16] Kim S. A., Choi H. C. (2012). Metformin inhibits inflammatory response via AMPK-PTEN pathway in vascular smooth muscle cells. *Biochemical and Biophysical Research Communications*.

[17] Wan X., Huo Y., Johns M. (2013). 5'-AMP-activated protein kinase-activating transcription factor 1 cascade modulates human monocyte-derived macrophages to atheroprotective functions in response to heme or metformin. *Arteriosclerosis, Thrombosis, and Vascular Biology*.

[18] Chinetti-Gbaguidi G., Colin S., Staels B. (2015). Macrophage subsets in atherosclerosis. *Nature Reviews Cardiology*.

[19] Labonte A. C., Tosello-Trampont A.-C., Hahn Y. S. (2014). The role of macrophage polarization in infectious and inflammatory diseases. *Molecules and Cells*.

[20] Qin Q., Niu J., Wang Z., Xu W., Qiao Z., Gu Y. (2012). Astragalus membranaceus inhibits inflammation via phospho-p38 mitogen-activated protein kinase (MAPK) and nuclear factor (NF)-*κ*B pathways in advanced glycation end product-stimulated macrophages. *International Journal of Molecular Sciences*.

[21] Miyata T., Inagi R., Iida Y. (1994). Involvement of *β*2-microglobulin modified with advanced glycation end products in the pathogenesis of hemodialysis-associated amyloidosis: induction of human monocyte chemotaxis and macrophage secretion of tumor necrosis factor-*α* and interleukin-1. *The Journal of Clinical Investigation*.

[22] Hattori Y., Hattori K., Hayashi T. (2015). Pleiotropic benefits of metformin: macrophage targeting its anti-inflammatory mechanisms. *Diabetes*.

[23] Vasamsetti S. B., Karnewar S., Kanugula A. K., Thatipalli A. R., Kumar J. M., Kotamraju S. (2015). Metformin inhibits monocyte-to-macrophage differentiation via AMPK-mediated inhibition of STAT3 activation: potential role in atherosclerosis. *Diabetes*.

[24] Ott C., Jacobs K., Haucke E., Navarrete Santos A., Grune T., Simm A. (2014). Role of advanced glycation end products in cellular signaling. *Redox Biology*.

[25] Cheng A., Dong Y., Zhu F., Liu Y., Hou F. F., Nie J. (2013). AGE-LDL activates toll like receptor 4 pathway and promotes inflammatory cytokines production in renal tubular epithelial cells. *International Journal of Biological Sciences*.

[26] Neacsu P., Mazare A., Schmuki P., Cimpean A. (2015). Attenuation of the macrophage inflammatory activity by TiO_2_ nanotubes via inhibition of MAPK and NF-*κ*B pathways. *International Journal of Nanomedicine*.

[27] Hu X., Chen J., Wang L., Ivashkiv L. B. (2007). Crosstalk among Jak-STAT, Toll-like receptor, and ITAM-dependent pathways in macrophage activation. *Journal of Leukocyte Biology*.

[28] Salminen A., Hyttinen J. M. T., Kaarniranta K. (2011). AMP-activated protein kinase inhibits NF-*κ*B signaling and inflammation: impact on healthspan and lifespan. *Journal of Molecular Medicine*.

[29] Huang B.-P., Lin C.-H., Chen H.-M., Lin J.-T., Cheng Y.-F., Kao S.-H. (2015). AMPK activation inhibits expression of proinflammatory mediators through downregulation of PI3K/p38 MAPK and NF-*κ*B signaling in murine macrophages. *DNA and Cell Biology*.

[30] Chen M., Zhang J., Hu F., Liu S., Zhou Z. (2016). Metformin affects the features of a human hepatocellular cell line (HepG2) by regulating macrophage polarization in a co-culture microenviroment. *Diabetes/Metabolism Research and Reviews*.

